# *In vitro* detection of marine invertebrate stem cells: utilizing molecular and cellular biology techniques and exploring markers

**DOI:** 10.3389/fcell.2024.1440091

**Published:** 2024-08-22

**Authors:** Fatemeh Mohajer, Arezoo Khoradmehr, Behnaz Riazalhosseini, Tuba Zendehboudi, Iraj Nabipour, Neda Baghban

**Affiliations:** ^1^ Student Research and Technology Committee, Bushehr University of Medical Sciences, Bushehr, Iran; ^2^ The Persian Gulf Marine Biotechnology Research Center, The Persian Gulf Biomedical Sciences Research Institute, Bushehr University of Medical Sciences, Bushehr, Iran; ^3^ The Pharmacogenomics Laboratory, Department of Pharmacology, Faculty of Medicine, University of Malaya, Kuala Lumpur, Malaysia; ^4^ Food Control Laboratory, Food and Drug Deputy, Bushehr University of Medical Sciences, Bushehr, Iran

**Keywords:** invertebrates, stem cells, marker, marine, regeneration, reproduction

## Abstract

Marine invertebrate stem cells (MISCs) represent a distinct category of pluripotent and totipotent cells with remarkable abilities for self-renewal and differentiation into multiple germ layers, akin to their vertebrate counterparts. These unique cells persist throughout an organism’s adult life and have been observed in various adult marine invertebrate phyla. MISCs play crucial roles in numerous biological processes, including developmental biology phenomena specific to marine invertebrates, such as senescence, delayed senescence, whole-body regeneration, and asexual reproduction. Furthermore, they serve as valuable models for studying stem cell biology. Despite their significance, information about MISCs remains scarce and scattered in the scientific literature. In this review, we have carefully collected and summarized valuable information about MISC detection by perusing the articles that study and detect MISCs in various marine invertebrate organisms. The review begins by defining MISCs and highlighting their unique features compared to vertebrates. It then discusses the common markers for MISC detection and *in vitro* techniques employed in invertebrate and vertebrates investigation. This comprehensive review provides researchers and scientists with a cohesive and succinct overview of MISC characteristics, detection methods, and associated biological phenomena in marine invertebrate organisms. We aim to offer a valuable resource to researchers and scientists interested in marine invertebrate stem cells, fostering a better understanding of their broader implications in biology. With ongoing advancements in scientific techniques and the continued exploration of marine invertebrate species, we anticipate that further discoveries will expand our knowledge of MISCs and their broader implications in biology.

## 1 Introduction

The diversity and phylogenetic range of marine invertebrates are unparalleled on Earth, from the simplest organisms like sponges and cnidarians to more complex creatures such as mollusks, crustaceans, echinoderms, and protochordate (Ballarin et al., 2018). A wide variety of cell and tissue types in invertebrates show remarkable flexibility in their shapes, structures, regenerative properties, proliferation processes, and cell lineage (Rosenfield et al., 1994; Rinkevich, 1999; Afshar et al., 2023; Baghban et al., 2023). Moreover, marine organisms produce different molecules including enzymes, biopolymers, bioactive compounds, and secondary metabolites which have applications in different fields (Ballarin et al., 2018; Keshavarz et al., 2021; Baghban et al., 2022; Carroll et al., 2022; Miri et al., 2022; Rasekh et al., 2023; Zare et al., 2023; Dehghani et al., 2024; Shamsian et al., 2024). For more than 150 years, due to these significant properties of marine invertebrates, they have served as laboratory models, illustrating various biological phenomena (Bellé et al., 2007; Ballarin et al., 2018). Besides, they also enable the production of numerous innovative bioactive molecules based on a kaleidoscope of marine invertebrate stem cell (MISC) types, many of which have significant potential for human health applications (Ballarin et al., 2018). These bioactive molecules will be used in various biological experiments, including stem cell research, further developing our understanding of marine invertebrate models (Ballarin et al., 2018).

Recent studies, have shown that marine invertebrates are ideal for stem cell research. These organisms provide various types and lineages of stem cells, which serve as significant models for studying stem cell biology (Ballarin et al., 2018). Stem cells, found in both embryonic development and organogenesis (embryonic stem cells), as well as tissue regeneration (adult stem cells), play pivotal roles in self-renewal and differentiation (Zhan, 2008). In multicellular organisms, stem cells have the unique ability to remain in an undifferentiated conditions and when needed, generate differentiated target cells (Varghese et al., 2009; Taichman et al., 2010; Ballarin et al., 2018). Instead of strictly interpreting stem cells as undifferentiated cells, it might be helpful to consider a broader interpretation that encompasses dedifferentiated cells in addition to stem cells (Kang et al., 2006; Xu et al., 2015; Ferrario et al., 2020), as defined by Post and Clevers’s (2019). Which places more emphasis on their ability to replace lost cells rather than on their morphological or molecular characteristics (Post and Clevers, 2019).

It is also noteworthy that trans-differentiation (a topic of great interest today when it comes to understanding “reprogramming” a cell) is found in both anatomically simple and “morphologically complex” invertebrates (Arenas-Mena, 2010; Knapp and Tanaka, 2012). There are many activities in which invertebrates can replace vertebrates, and invertebrates can also offer new perspectives on the function, properties, and evolution of MISCs (Ballarin et al., 2018). Research on MISCs contributes to our understanding of various biological processes, including cellular growth and differentiation, aging, and senescence, as well as regeneration and budding that occur in marine invertebrates (Ballarin et al., 2018). Therefore, effective detection approaches for these cells become more significant. In this regard, this review summarizes the most common techniques used to detect MISCs based on previous studies on stem cell investigation, and the types of detected stem cell markers in marine invertebrates.

**FIGURE 1:**
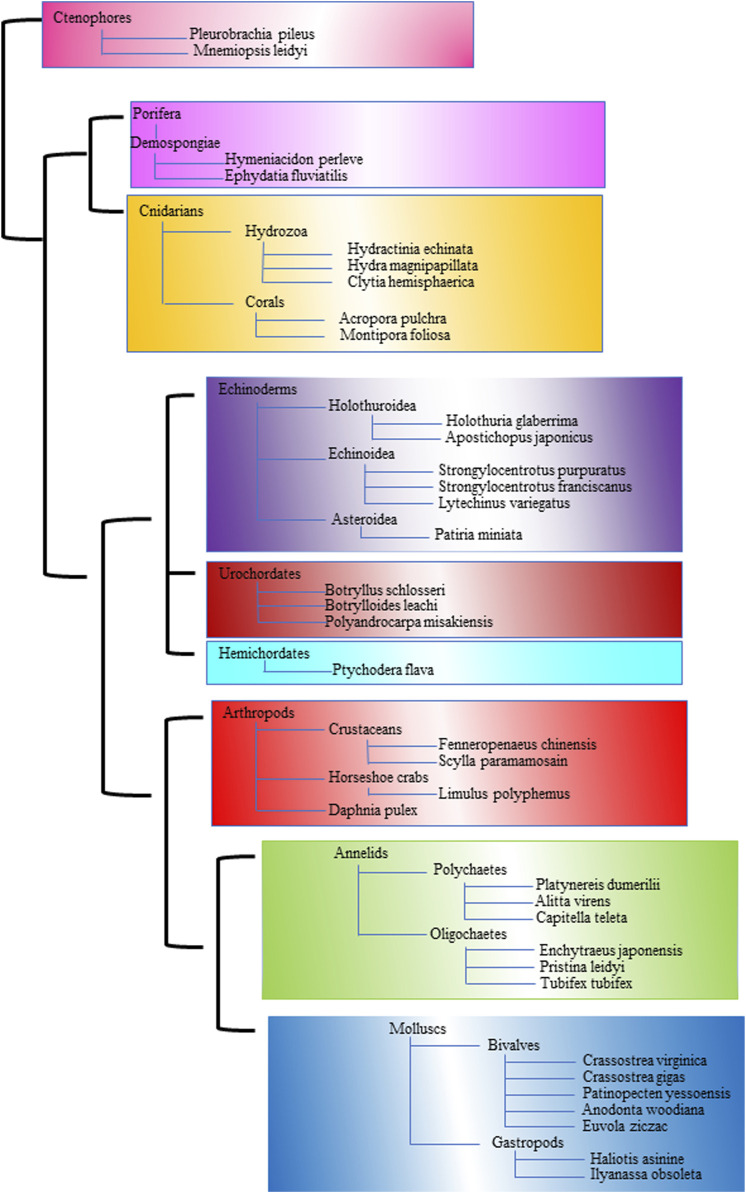
The phylogenetic tree illustrates the evolutionary relationships among various marine and terrestrial invertebrates. A distinct color represents each major group.

Figure 1 is a comprehensive phylogenetic tree to elucidate the evolutionary relationships among diverse marine and terrestrial invertebrates. This tree includes major invertebrate groups such as Arthropods, Crustaceans, Molluscs, Bivalves, Gastropods, Annelids, Polychaetes, Echinoderms, Cnidarians, Sponges, Hemichordates, and Ctenophores (Zhang et al., 2021). By incorporating a range of invertebrates, this phylogenetic tree aims to depict the complex web of evolutionary paths these organisms have followed. The construction of this tree is based on both morphological characteristics and molecular data, providing a robust framework for understanding the evolutionary history and relationships among these diverse groups. Such a phylogenetic tree not only highlights the shared ancestry and divergence of these invertebrates but also aids in the identification of unique evolutionary traits and adaptations that have arisen in response to different ecological niches. This visual representation is a valuable tool for comparative studies, helping unravel the evolutionary processes that have shaped the rich biodiversity of invertebrates in marine and terrestrial environments.

## 2 Marine invertebrates stem cells (MISCs)

Stem cells are believed to exist in various marine organisms, ranging from simple species like sponges to more complex echinoderms and crustaceans (Ballarin et al., 2018). While adult stem cells are prevalent across marine invertebrate phyla, extensive examination has primarily focused on porifera, cnidaria, and platyhelminths. Despite this, our understanding of Marine Invertebrate Stem Cells (MISCs) is limited, especially when compared to vertebrates, mammals, and terrestrial invertebrates (Rinkevich et al., 2009).

MISCs exhibit distinctive cytomorphological features, being smaller in size with a higher nucleo-cytoplasmic ratio, basophilic staining, and rounded shapes (Cima et al., 2001). Hemocytes in Botryllus, as well as neoblasts in many species of oligochaete worms and *Enchytraeus japonensis*, display stem cell characteristics (Randolph, 1892; Oka and Watanabe, 1957; Rinkevich et al., 2007; Rinkevich et al., 2008; Yoshida-Noro and Tochinai, 2010). Unlike vertebrates, MISCs have a widespread distribution throughout the body, lacking clear distinctions between germ and somatic stem cell lineages (Rinkevich and Matranga, 2009; Sköld et al., 2009). In some marine invertebrates like Platyhelminthes and hydra, stem cells can differentiate into both germ and stem lineages, allowing germline establishment in the adult phase (Hwang et al., 2007). Notably, germ-stem cells in marine invertebrates have been observed to trans-differentiate into somatic adult stem cells in specific regeneration scenarios (Gremigni and Puccinelli, 1977; Gremigni, 1981). Marine invertebrates exhibit pluripotency and totipotency, differentiating into cell lines from multiple germ layers, including the germline, a unique trait when compared to the unipotent or oligopotent nature of vertebrate stem cells (Rinkevich et al., 2009). MISCs can proliferate, differentiate, and migrate to various tissues throughout adulthood, playing pivotal roles in various biological processes. These processes include senescence, delayed senescence, longevity, whole-body regeneration, asexual reproduction methods like budding, fragmentation, gemmule-hatching, indeterminate growth, fission, and torpor phenomena. Additionally, MISCs contribute to unique stemness systems in marine invertebrates, without forming tumors (Shimomura et al., 1962). Ongoing research into dedifferentiation and the spontaneous reacquisition of stem cell phenotypes, has potential implications in reprogramming, as seen with the addition of Yamanaka’s factors in mammalian cultures. Therefore, MISCs, are central to the developmental biology phenomena of marine invertebrates (Okita et al., 2007).

## 3 MISC identification

Scientists use stem cell markers, which are genes and their protein products, to identify and isolate stem cells, including MISCs (Pazhanisamy, 2013). These markers can be specific proteins on the cell surface or intracellular proteins within the cell (Nguyen et al., 2010). Using antibodies, either monoclonal or polyclonal, to identify stem cell lineages is one of the strategies to assign the stemness phenotype based on an analysis of surface molecules and intracellular markers (Cai et al., 2005).

In recent years, there has been a surge in the development and utilization of various methods for detecting and characterizing stem cell markers. These techniques, encompassing flow cytometry, immunofluorescence/immunocytochemistry, protein array, real-time RT-PCR, and Western blot, have significantly advanced our understanding of stem cell biology. Their continued development promises to further enhance stem cell research and contribute significantly to the field of regenerative medicine.

### 3.1 Markers used to identify MISCs

Accurate identification of stem cells relies on specific molecular markers, which are recognized by their distinct expression patterns rather than their functions (Cai et al., 2005). Although no single marker is sufficient to identify a specific stem cell, several molecules have been identified, such as Vasa, Sox2, Oct3/4, Nanog, alkaline phosphatase, and telomerase activity (Sköld et al., 2009; Zito and Matranga, 2009). Additionally, common “stemness” genes like Piwi, Nanos, PCNA, and Aldh are utilized in both vertebrate and invertebrate stem cells (Rinkevich et al., 2009). Studies reveal molecular similarities between ascidian and mammalian stem cells, including Aldh, alkaline phosphatase, PL10, and PCNA. Genes related to the Germline/Multipotency program (GMP) like Piwi, Vasa, Nanos, and PL10 are highly conserved across various metazoan species (Juliano et al., 2010). The markers listed in Table 1 are frequently employed for identifying MISCs and will be further elaborated in more detail in the following sections.

**TABLE 1:** Stem cell markers in marine invertebrates: a closer look at locations and functions.

Marker	Location	Function
Aldehyde dehydrogenase	Cytoplasm, Mitochondria, Endoplasmic Reticulum, Nucleus (Jiang et al., 2009)	Essential for safeguarding stem cells from harmful internal and external aldehydes and facilitating their differentiation (Kang et al., 2002), as well as playing a vital role in the self-renewal process of stem cells (Zhou et al., 2019)
Alkaline phosphatase	Cytoplasm (Isaeva et al., 2003; Cavelier et al., 2017)	Engaged in a diverse range of biological processes, such as metabolite processing, transportation, and secretion, as well as playing crucial roles in stem cell functions and biomineralization (Buchet et al., 2013). It is commonly regarded as a significant indicator of both stem cells and germline cells
Bmi1	Nucleus (Haupt et al., 1993; Jacobs et al., 1999)	Bmi1, known as B-cell-specific Maloney murine virus integration site, belongs to the Polycomb group of transcription repressors, which controls epigenetic gene silencing. It plays a critical role in supporting the maintenance and self-renewal of stem cells (Siddique and Saleem, 2012)
Brdu	Nucleus (Mashanov et al., 2017a)	Synthetic nucleoside analog of thymidine that is often used as a marker for detecting DNA synthesis in cell proliferation and tissue regeneration processes (Taupin, 2007; Benton et al., 2014)
c-Myc	Nuclear (Takahashi et al., 2007; McConnell and Yang, 2010)	A Yamanaka factor, with the ability to regulate the transcription of approximately 15% of all protein-coding genes, plays a crucial role in a diverse array of biological processes in mammals (Mahani et al., 2013). It also governs growth and cell proliferation (Gallant, 2006)
Edu	Nuclear (Benton et al., 2014)	EdU is a thymidine analog used as a marker to detect DNA synthesis in proliferating cells. It has been employed as a tool to study cell proliferation and regeneration processes (Qu et al., 2011)
Grimp	Nucleus (Takeo et al., 2009)	One of the crucial genes that plays a vital role in the initial cell division and growth (Takeo et al., 2009)
Klf	Nucleus (Durham et al., 2016)	Klf proteins are transcription factors characterized by zinc-finger motifs. Klf2 and Klf4 serve as tumor suppressor genes, actively inhibiting cell growth, DNA synthesis, and cell cycle progression. (Takahashi et al., 2007; McConnell and Yang, 2010)
Lgr5	Transmembrane (de Lau et al., 2014)	Lgr5 (Leucine-rich-repeat-containing G-protein-coupled Receptor) functions as an R-spondin receptor. Upon binding to its ligand, it amplifies the Wnt signal by inhibiting the Wnt negative feedback loop. This receptor is involved in various aspects of stem cell maintenance (Glinka et al., 2011; de Lau et al., 2014)
Nanog	Nucleus (Chambers et al., 2003)	The transcription factor described is a crucial component of the core set of factors, along with OCT4 and SOX2, responsible for establishing and maintaining self-renewal and pluripotency in embryonic stem cells (ESCs) (Mitsui et al., 2003)
Nanos	Nucleus (Wang et al., 2007)	In many marine invertebrates, nanos is primarily expressed in germline cells, where it acts as a translational inhibitor to regulate the development and maintenance of germ cells (Rinkevich et al., 2022)
Oct-4	Nucleus (Niwa et al., 2000; Cavaleri and Schöler, 2003)	This transcription factor plays a central role in the regulatory network, responsible for both maintaining the pluripotent state and suppressing genes that drive cell differentiation (Boyer et al., 2005; Park et al., 2013)
PCNA	Nucleus (D’Angelo et al., 2012)	An accessory protein that assists DNA polymerases during the replication process (D’Angelo et al., 2012)
Piwi	Nucleus (Hutvágner and Zamore, 2002)	Serve as epigenetic modulators of stem cells (Hatfield et al., 2005), participating in RNA interference which leads to mRNA degradation and specific gene silencing (Hutvágner and Zamore, 2002). Impact processes like germline determination, germline maintenance, gametogenesis, and stem cell self-renewal (Thomson and Lin, 2009)
PL10	Nucleus (Yoshizaki et al., 2000; Mochizuki et al., 2001)	Its function is associated with RNA metabolism and translation regulation. It plays a role in various cellular processes, including RNA splicing, ribosome biogenesis, and translation initiation (Kozin and Kostyuchenko, 2015)
Sox2	Nucleus (Chambers et al., 2007)	Sox2 plays a crucial role in embryonic development, stem cell maintenance, and tissue-specific functions in various organisms, making it a key transcription factor in the field of developmental and stem cell biology (Jemaà et al., 2014; Cavelier et al., 2017)
Telomerase activity	Mostly in nucleus (Blackburn, 1991)	The level of telomerase activity in stem cells is indicative of their ability to self-renew (Morrison et al., 1996) and their potential for proliferation (Lang et al., 2004)
Vasa	Nucleus (Milani et al., 2015; Milani et al., 2017)	Its function is critical for germline development and maintenance (Rinkevich et al., 2009)

#### 3.1.1 Aldehyde dehydrogenase

Aldehyde dehydrogenase (ALDH) plays diverse roles in cells, including detoxification of aldehydes, retinoic acid production, biomolecule synthesis, promoting cell survival, and hormonal interactions (Marchitti et al., 2008; Zhou et al., 2019). There are 24 ALDH protein families with varied functions, that are crucial for drug resistance and stem cell protection (Moreb, 2008; Jackson et al., 2011; Vasiliou et al., 2013). ALDH activity is essential for adult stem cell self-renewal, and is closely linked to stemness and differs during hypoxia in species like shrimp *Fenneropenaeus chinensis* (Jiang et al., 2009). ALDH also contributes to ascidian species development and has been studied in somatic tissue of marine species like *Limulus polyphemus* and *Crassostrea virginica* for stress adaptation (Dragolovich and Pierce, 1994; Perrino and Pierce, 2000; Sköld et al., 2009).

It is important to note that while ALDH is vital for transforming progenitors cells into mature cells, its specificity for stem cells is limited (Vassalli, 2019). Certain ALDH isozymes may not maintain stemness, emphasizing the need to identify stemness-related ALDH isozymes and their roles in renewing and differentiating cells (Moreb, 2008). ALDH has been detected in the asexual buds of the ascidian Polyandrocarpa misakiensis. This was found using the ALDEFLUOR assay, aiding in ALDH detection through flow cytometry (Storms et al., 1999; Zhou et al., 2019).

#### 3.1.2 Alkaline phosphatase

Alkaline phosphatases (ALPs) are essential metalloenzymes found in various life forms and have been studied since 1954, serving as markers for germ cells in mice (Chiquoine, 1954; Cavelier et al., 2017). These enzymes are predominantly present in stem cells and germline cells, contributing to metabolite transport, secretion, stem cell maintenance, biomineralization, and tissue regeneration in specific animals (Buchet et al., 2013). In human embryonic stem cells, ALP activity indicates pluripotency and diverse differentiation potential. Notably, ALP activity is not limited to mammals but extends to diverse organisms, including oysters and invertebrates like ascidians, parasitic crustaceans, hydrozoans, planarian neoblasts, and crustaceans (Osborne and Miller, 1963; Millan and Manes, 1988; Extavour and Akam, 2003; Shukalyuk et al., 2005; O’Connor et al., 2008; Isaeva et al., 2011). This underscores ALP’s wide-ranging importance in stem cell functions. For instance, in oysters, robust ALP activity in reproductive areas and metabolic processes has been detected (Bevelander, 1952). Similarly, ALP activity in Polyascus polygenea’s stolons, primordial cells, and the polychaete Pomatoceros lamarckii highlights its role in stem cell-related functions (Isaeva et al., 2003; Szabo and Ferrier, 2014).

#### 3.1.3 BMI1

BMI1, a transcription repressor in the Polycomb family of transcription factors, is essential for governing gene expression and preserving stem cell reservoirs, ensuring self-renewal, and maintaining these critical cell populations. Its primary role is to prevent premature senescence, ensuring stem cell longevity and functionality (Siddique and Saleem, 2012). BMI1’s presence in mice suggests that it plays a crucial role in sustaining somatic stem cells throughout the body and is associated with oncogenic potential (Schmitt, 2003). Moreover, BMI1 is not limited to mice; it also plays a role in self-renewal in various stem cell populations, including adult hematopoietic stem cells (HSCs), peripheral neural stem cells, and central nervous system neural stem cells (Haupt et al., 1993; Jacobs et al., 1999). In non-mammalian species like the sea cucumber *Holothuria glaberrima*, BMI1 serves as an analogue to mammalian intestinal stem cell markers, indicating its conserved role in regulating stem cells across different organisms (Molofsky et al., 2003; Park et al., 2003; Mashanov V. et al., 2017a). This underscores the wide-reaching importance of BMI1 in stem cell biology.

#### 3.1.4 BrdU

BrdU (5-bromo-2′-deoxyuridine) labeling is widely used to identify cell divisions and serves as a valuable marker for DNA synthesis and cell division rates (Mashanov et al., 2017b). It is particularly effective for tracking actively dividing cells during the S-phase of the cell cycle, although caution is needed due to potential incorporation during DNA repair processes (Dolbeare and Selden, 1994; Taupin, 2007; Benton et al., 2014). In marine organisms like *Hymeniacidon perleve*, BrdU incorporation in archaeocytes (toti/multipotent stem cells), along with increased PCNA expression and telomerase activity, indicates DNA synthesis and cell proliferation (Sun et al., 2007).

Cnidarians like *Hydractinia echinata* and *Clytia hemisphaerica* utilize BrdU in pulse-chase assays, shedding light on mitotic activity and cellular self-renewal dynamics (Müller et al., 2004; Denker et al., 2008). In the hatchings and adults marine platyhelminth *Macrostomum* sp., BrdU incorporation reveals neoblast migration, renewal capabilities, and differentiation into various somatic and germ cells, enhancing understanding of stem cell dynamics (Ladurner et al., 2000; Schärer et al., 2004). BrdU labeling proves versatile in exploring cell proliferation, stem cell behavior, and tissue renewal in diverse marine organisms, advancing our understanding of cellular processes and promising further research in regenerative biology and developmental studies, with necessary acknowledgment of its limitations for accurate interpretation.

#### 3.1.5 c-Myc

The c-Myc gene, a pivotal transcription factor, regulates essential cellular functions such as proliferation, growth, and apoptosis (Lehr, 2003). In both invertebrates and vertebrates, Myc proteins play a crucial role in managing cell growth and proliferation, highlighting their conserved regulatory function across species (Gallant, 2006; Takahashi et al., 2007; Morita et al., 2009; McConnell and Yang, 2010; Mahani et al., 2013). For instance, marine invertebrates like the sea cucumber *H. glaberrima* possess a c-Myc gene family, with elevated Myc levels correlating with tissue dedifferentiation and regeneration, emphasizing Myc’s importance in tissue growth and repair in marine organisms (Mashanov et al., 2015).

Studies on the sea star species Asterias vulgaris have identified c-myc proto-oncogene expression as a sensitive indicator for assessing mitotic activity in the testicular germinal epithelium, shedding light on growth and reproduction regulatory mechanisms in marine organisms (Marsh and Walker 1995).

Understanding proto-oncogenes like c-Myc in marine organisms provides crucial insights into the molecular mechanisms driving growth, development, and regeneration. Exploring Myc-related pathways reveals fundamental processes enabling marine organisms to adapt and respond to environmental changes, including tissue repair and regeneration. These insights not only expand our knowledge of fundamental biological processes but also have potential implications for regenerative medicine and stem cell research in humans. The universal functions of Myc across species emphasize the importance of studying marine invertebrates as model organisms, enriching our understanding of these processes.

#### 3.1.6 EdU

EdU (5-ethynyl-2′-deoxyuridine) is a groundbreaking thymidine analog used for DNA labeling in actively dividing cells. Its unique feature, employing fluorescent azides for detection, eliminating the need for DNA denaturation and providing deep tissue penetration for *in vivo* studies, making it invaluable for tracking stem cells and cell proliferation in living organisms (Lin et al., 2010; Qu et al., 2011). EdU labeling has provided crucial insights into cell proliferation patterns in various organisms. For instance, in *Aeolosoma viride* (Chen et al., 2020) and *Pristina leidyi* (Bely and Sikes, 2010) distinct proliferation patterns highlighted by EdU labeling led to variations in blastema size during regeneration (Bely and Sikes, 2010). In *Playtnereis dumerilli*, EdU pulse-labeling revealed unique primordial germ cell (PGC) clusters anterior to the mesodermal posterior growth zone, demonstrating its specificity in detecting cell populations (Rebscher et al., 2012; Zattara and Bely, 2013; Ćorić et al., 2022). EdU has illuminated cell proliferation during adult regeneration in species like the Sea Anemone *Nematostella vectensis* and *Ptychodera flava*, showing its applicability in studying tissue regeneration and cell dynamics (Amiel et al., 2015; Luttrell et al., 2018).

In hydra, EdU labeling indicated the presence of slow-cycling adult stem cells in early multicellular organisms, emphasizing its role in studying stem cell biology and evolution (Govindasamy et al., 2014). Additionally, EdU assays conducted on hammer coral (*Fimbriaphyllia ancora*) elucidated somatic cell proliferation during gametogenesis which advanced our understanding of coral reproduction (Chiu et al., 2021). Furthermore, experiments with Pleurobrachia pileus demonstrated EdU’s ability for DNA-labeling and long-term retention. This helped link stem cell concentrations with gene expression areas, emphasizing its role in pinpointing stem cell niches and connecting somatic stem cells to gene regulation (Alié et al., 2011). EdU’s non-invasive *in vivo* detection method, coupled with its versatility, makes it a crucial tool for studying cell proliferation, stem cell biology, and regeneration across diverse organisms. As research progresses, it is expected that EdU will play a pivotal role in enhancing our understanding of stem cells within multicellular development and regeneration contexts.

#### 3.1.7 Grimp

The Grimp gene, which has been identified in *E. japonensis*, serves as a crucial regulator of cell proliferation, particularly in multipotent mesodermal cells (neoblasts), highlighting its significant role in the early stages of regeneration. Its impact extends to various mesodermal cells beyond neoblasts, emphasizing its vital function in regulating mesodermal cell proliferation during the onset of the regenerative process. When the Grimp mRNA is suppressed, it disrupts cell proliferation in the mesoderm, and inhibits anterior structure differentiation, indicating the pivotal role of Grimp in mesodermal regeneration in *E. japonensis* (Takeo et al., 2009).

It is important to understand the function of the Grimp gene in order to advance regeneration and stem cell biology. Its promotion of neoblast proliferation provides insights into the mechanisms governing tissue repair and regrowth, considering the crucial role of neoblasts in regeneration across organisms (Yoshida-Noro and Tochinai, 2010). Exploring non-neoblast Grimp-expressing cells in E. japonensis mesoderm could offer insights into complex mesodermal cell proliferation and differentiation during regeneration. Furthermore, investigating Grimp-related regulatory networks may deepen our understanding of the molecular mechanisms driving tissue regeneration and repair. Grimp research stands as a promising potential in regenerative biology, with implications for enhancing tissue repair and regeneration in various organisms, including humans. Exploring its mechanisms could lead to innovative approaches in regenerative medicine and illuminate fundamental principles of stem cell biology (Takeo et al., 2009).

#### 3.1.8 Klf

The Krüppel-like factor (Klf) gene family is known for its involvement in fundamental processes like stem cell renewal, pluripotency, and differentiation. This gene family consists of zinc-finger transcription factors, specifically KLF2 and KLF4. These genes exhibit tumor suppressor properties, inhibiting cell growth, DNA synthesis, and cell cycle progression, with KLF4 having dual capabilities in promoting cell survival and counteracting c-Myc-induced programmed cell death (Takahashi et al., 2007; McConnell and Yang, 2010).

While most research on Klf genes has focused on select bilaterian animals, there is growing interest in their potential evolutionary. Studies on the ctenophore *Mnemiopsis leidyi* have revealed Klf genes in stem cells that regulate proliferation in developmental stem cell niches, suggesting their contribution to the stem lineage leading to Metazoa, emphasizing their role in governing stem cell proliferation in metazoans (Presnell, 2017). Sea urchin genomes contain multiple Klf genes, and Klf1/2/4 have been identified as orthologs of Holothuria glaberrima, indicating their conservation across echinoderms (Materna et al., 2006; Mashanov et al., 2015).

In *Schmidtea mediterranea* planarians, the Klf4-like gene (klf4l) has been studied for its role in maintaining germ cells and yolk cells. Knocking down Klf4l impedes these cell types, highlighting its crucial role in their regulation. Moreover, Klf4 marks expression in PGCs and presumptive germline stem cells (GSCs) of planarians, emphasizing its significance in germ cell regulation. In the sea cucumber Apostichopus japonicus, Aj-klf13 plays a pivotal role in intestinal regeneration, showcasing diverse roles of KLFs in tissue regeneration and repair across organisms (Chen et al., 2020b; Issigonis et al., 2022).

Hydra polyps as a simple multicellular animal, demonstrate intricate KLF roles, particularly in interlineage communication and cell type-specific functioning within three stem cell populations. Overall, the multifaceted roles of the Klf gene family, along with their conservation across metazoans, underscore their importance in developmental processes, stem cell regulation, and tissue regeneration. Deeper exploration of KLF molecular mechanisms and evolution is expected to yield new insights into stem cell biology and regenerative medicine, offering innovative avenues for future therapeutic interventions and treatments (Hemmrich et al., 2012).

#### 3.1.9 Lgr5

The trio of receptors, Lgr5, Lgr4, and Lgr6, which belong to the LGR family, play a pivotal role in adult stem/progenitor cells. These receptors, characterized by a large extracellular domain with leucine-rich repeat (LRR) units, are regulated by Wnt signaling and are crucial for cellular growth and division. Dysregulated Wnt activation, often due to mutations, leads to strong Lgr5 expression in various cancers (de Lau et al., 2014).

In sea cucumber *H. glaberrima*, homologs of mammalian Lgr5 (Lgr4/5/6) are expressed in peritoneal cells, suggesting their candidacy as gut mesothelium stem cells. Both Lgr5 and Bmi1 stem cell gene markers are found in epithelial and peritoneal cells of the digestive tube, highlighting their role in tissue maintenance and regeneration (de Lau et al., 2014). Investigating Lgr4/5/6 interactions with R-spondins in sea cucumbers can shed light on stem cell self-renewal and tissue repair pathways, underscoring evolutionary stem cell regulatory conservation (Mashanov et al., 2017a).

Advancements in understanding LGR roles, such as Lgr5, Lgr4, and Lgr6, hold therapeutic potential in regenerative medicine and cancer therapy. Exploring LGR signaling pathways and mechanisms enriches stem cell biology knowledge, with implications for human health. The identification of Lgr4/5/6 in sea cucumbers and their expression in peritoneal cells hints at their diverse roles in stem cell regulation and tissue balance, promising novel therapies in regenerative medicine driven by exciting discoveries (Mashanov et al., 2017b).

#### 3.1.10 Nanog

Nanog, a vital transcription factor, plays a key role in maintaining pluripotent cells and embryonic stem cells’ (ESCs) self-renewal capacities. In marine organisms like Hydra and Nematostella, transcription factors including Nanog, Oct-4, and Klf4 regulate stemness and robust proliferation in vertebrate ESCs. Despite the challenges associated with influencing specific genes in marine invertebrates due to limited understanding of growth factor gene expression, the sea urchin *S. purpuratus* possesses conserved genes like SpOct and a nanog homolog that are associated with pluripotency. Exploration of DNA sequences has led to the identification of a nanog gene homolog in marine invertebrates, particularly within the sea urchin genome, highlighting its significance in understanding stem cell regulation and pluripotency (Char et al., 1993; Yuh et al., 1998; Chambers et al., 2003; Mitsui et al., 2003; Rodda et al., 2005; Odintsova et al., 2007; Watanabe et al., 2009; Ballarin et al., 2022).

#### 3.1.11 Nanos

Nanos family proteins, which characterized by conserved CCHC zinc fingers, play crucial roles in the development of somatic and germ line cells across various Metazoa species. While they are primarily associated with germline functions, nanos genes exhibit diverse expression patterns, suggesting multiple roles in embryonic and larval development (Curtis et al., 1997; Extavour and Akam, 2003; Wang et al., 2007; Rinkevich et al., 2022). They contribute to the germline multipotency program (GMP) alongside genes like piwi and vasa, and are served as robust molecular markers for multipotent cells. In certain contexts, such as regenerative blastema, *de novo* nanos expression may indicate local cell dedifferentiation (Juliano et al., 2010; Nikanorova et al., 2020; Kostyuchenko and Kozin, 2021).

Nanos expression patterns vary in embryonic and larval development - among various marine organisms. For instance, in *P. ornatus*, nanos expression increases in day 3 embryos, aligning with gastrula-specific gene expressions (Helluy and Beltz, 1991). In *E. sinensis* embryos, genes like Nanos are upregulated during early developmental stages (Wang et al., 2020).

The transcripts of CapI-vasa and CapI-nanos are present in developing gametes of mature adults, and their expression is observed across embryonic, larval, and juvenile stages. These transcripts are found in various somatic tissues, with broad expression during early cleavage stages. As gastrulation progresses, their expression becomes prominent in areas such as the presumptive brain, mesodermal bands, and developing foregut. Researchers have used CapI-nanos and CapI-vasa as markers to identify potential PGCs in larvae, initially forming small bilateral clusters in segment four and later consolidating into a single cluster in late larval stages (Dill and Seaver, 2008)

Nanos-related genes are crucial in lophotrochozoans like *Helobdella robusta* and *P. dumerilii*, expressed in germ cells and PGCs (Kang et al., 2002; Zelada González, 2005; Rebscher et al., 2007). In these species, Nanos expression extends to both germ cells and somatic tissues (Kang et al., 2002; Zelada González, 2005). Knock down experiments in *Ilyanassa obsoleta* demonstrate Nanos’s importance in mesodermal and endodermal tissue development (Rabinowitz et al., 2008).

*Patinopecten yessoensis* possesses two Nanos family genes, PyNanos1 and PyNanos2/3, expressed in distinct gonadal cells. In the Tritia obsoleta, Nanos expression is limited to the 4d mesoderm lineage, and it is vital for mesodermal and endodermal tissue preservation. Haliotis asinine displays varied HasNanos expression, which is crucial in embryonic development and is localized in the dorsal quadrant (Rabinowitz et al., 2008; Liu et al., 2022).

During trochophore development in Haliotis asinine, Nanos is found in putative mesodermal bands and PGCs. In leeches, polychaetes (*Platynereis dumerilii* and *Capitella* sp. *I*), Nanos is integral to germ cell and PGC formation (Kang et al., 2002; Zelada González, 2005; Dill and Seaver, 2008; Kranz et al., 2010; Ponz-Segrelles et al., 2018). Nanos is expressed diversely in somatic tissues, including ectodermal stem cells in leeches, and brain, foregut, mesodermal bands, and growth zones in *P. dumerilii* and *Capitella* sp. *I*. Furthermore, Pdu-nanos is involved in the regeneration process of *P. dumerilii* worms (Kang et al., 2002; Zelada González, 2005; Planques et al., 2019).

#### 3.1.12 Oct-4

The Oct4 gene, a member of the POU protein family, is essential for establishing and maintaining pluripotent stem cells, governing key target genes to preserve pluripotency and inhibit differentiation. Oct4 downregulation during cell differentiation is harnessed for reprogramming somatic cells into pluripotent stem cells (Pesce et al., 1998; Niwa et al., 2000; Cavaleri and Schöler, 2003; Boyer et al., 2005; Anderson et al., 2007; Takahashi et al., 2007; Park et al., 2013).

Oct4-like POU domain gene expression is observed in pluripotent interstitial cells (I cells) in hydrozoan cnidarians *Hydractinia stolons* and in sea urchins. In the sea cucumber *H. glaberrima*, Oct1/2/11 shows homology with mammalian Oct4 (Eddy, 1976; Frank et al., 2009; Range and Lepage, 2011; Mashanov, et al., 2015).

Introducing the Oct4-like transcription factor Polynem (Pln) into Hydractinia’s epithelial cells transforms them into stem cells, leading to neoplasm development. Oct4 induces MALAT1 transcription, promoting cell proliferation and motility, and regulates Dnmt1 activity. Strong Oct4 expression is found in mature ovaries of mud crabs, particularly in the eyestalk of female crabs. Oct4/Sox9 interaction enhances SpVih expression, while interference with Oct4 and Sox9 reduces SpVih expression and increases SpVtg expression in ovaries and hepatopancreas. GST pull-down experiments support the interactions between Oct4 and Sox9, emphasizing their role in SpVih regulation (Plickert et al., 2012; Jen et al., 2017; Wu et al., 2018; Liao et al., 2020).

#### 3.1.13 PCNA

The Proliferating Cell Nuclear Antigen (PCNA) serves as a crucial biomarker for active cell division and plays essential roles in DNA replication, damage repair, and cell cycle progression. Studies in Cnidarian species like *Montipora foliosa* and *Acropora pulchra* have utilized PCNA protein expression to investigate mitotic activity, indicating its evolutionary conservation (D’Angelo et al., 2012). In sponges, specifically in archaeocytes of *Hymeniacidon*, remarkably high PCNA levels were observed (Sun et al., 2007).

Oysters such as *Crassostrea gigas* exhibited elevated CgPCNA mRNA transcripts in various tissues, including gonads, gills, and haemolymph (Yu et al., 2022). Invertebrate stem cells, identified through markers like germinal granules and alkaline phosphatase, demonstrated specific PCNA activity (Isaeva et al., 2008).

Additionally, the introduction of recombinant protein CgAstakine in oysters increased CgPCNA protein abundance in agranulocytes and gills, implicating its role in hematopoiesis (Yu et al., 2022). Furthermore, spatial distribution studies using BrdU-PCNA immuno-detection, neuron-specific staining, and electron microscopy provided insights into mesenchymal stem cells and neurons in the striated adductor muscle of scallops (Sun et al., 2021).

#### 3.1.14 Piwi

The Piwi-like genes, part of the Argonaute protein subfamily, are crucial in stem cell epigenetics and RNA interference across various biological systems. They participate in mRNA degradation and gene silencing, impacting germline development and stem cell regulation (Hutvágner and Zamore, 2002; Hatfield et al., 2005; Thomson and Lin, 2009; Giani et al., 2011). Studies on sea urchin have highlighted their role in spine and tube foot regeneration, indicating multipotent progenitor cells’ involvement (Juliano et al., 2006; Karthik et al., 2014; Bodnar and Coffman, 2016).

Piwi gene expression is predominant during gametogenesis and embryonic development, exhibiting varying patterns across species (Deng and Lin, 2002; Carmell et al., 2007; Houwing et al., 2008; Wang and Reinke, 2008; Solana et al., 2012; Juliano et al., 2014). Across diverse organisms, Piwi influences regeneration, with examples in Botrylloides species and cnidarians like jellyfish (Seipel et al., 2003; Juliano et al., 2006; Frank et al., 2009; Alié et al., 2011; Brown and Swalla, 2012; Vickers, 2017).

*Capitella teleta*’s Piwi genes have dual roles in somatic and germline stem cells (Giani et al., 2011). Piwi’s presence in stony coral, sea anemone, jellyfish, and hydroids influences germ cell formation (Rabinowitz and Rinkevich, 2011).

Specific Piwi-like genes in species like *Lygdamis sanguineus* play tissue-specific roles, particularly in early regeneration. While they are typically involved in silencing, there are unique cases such as flatworms and acoels expressing Piwi-like genes in somatic stem cells. Annelid species exhibit Piwi-like gene expression in posterior growth zone cells and gonadal stem cells (Extavour et al., 2005; Rebscher et al., 2008; Alié et al., 2011; Leclère et al., 2012; Shikina et al., 2012; Weigert et al., 2013; Hartung et al., 2014; Kozin and Kostyuchenko, 2015; Shikina et al., 2015; Xu and Sun, 2020).

#### 3.1.15 PL10

The Vasa and PL10 homologs, both are members of the DEAD-box family, have a close relationship (Yoshizaki et al., 2000; Mochizuki et al., 2001). These genes are present in various organisms such as sponges, Hydra, and planaria, which are belonging to invertebrates and non-mammal vertebrates (Mochizuki et al., 2001). In these groups, Ded1/DDX3 genes are often referred to as PL10. Notably, PL10 plays a significant role in spermatogenesis and differentiation in invertebrates like Hydra and planarians. While PL10 is typically the only member of the subfamily in non-mammal animals and some noneutherian mammals, it does show a distinct localization pattern in sponges and cnidarians but has been less studied in vertebrates.

Research on zebrafish germ cells has revealed a high transcription of the PL10-homologous gene (pl10a) using RNA *in situ* hybridization. This suggests that PL10 has an important function in maintaining the undifferentiated state of PGCs and multipotent stem cells among invertebrates like the nereid polychaete *Alitta virens* (Kozin and Kostyuchenko, 2015). Furthermore, PL10 expression has been identified in the germlines of both male and female *Pleurobrachia pileus* (Alié et al., 2011).

PL10, a DEAD-box RNA helicase closely related to Vasa, and Bruno, an RNA-binding protein with RRM domains, play crucial roles in cellular processes. In mammals, PL10 (also known as DDX3) is indispensable for spermatogenesis, ensuring normal cellular differentiation during this process (Abdelhaleem, 2005). Expression of PL10 and Bruno genes is observed in planarian neoblasts (Shibata et al., 1999; Guo et al., 2006), while Piwi, Vasa, and PL10 genes are co-expressed in the posterior growth zone of Platynereis larvae (Rebscher et al., 2007), playing a role in somatic and germinal stem cell activity. PL10 is expressed in progenitor cell clusters, gonads, and the posterior growth zone of *C. teleta*, indicating a potential dual role in somatic and germline cells (Seaver and de Jong, 2021).

#### 3.1.16 Sox2

Sox2, a stem cell-specific transcription factor, possesses a high mobility group (HMG) DNA binding domain that interacts with the 5-CATTGTT-3 consensus motif (Chambers et al., 2007) Functioning within the core regulatory network, Sox2 maintains pluripotency and suppresses differentiation-inducing genes in stem and precursor cells (Boyer et al., 2005; Park et al., 2013; Liu and Nonomura, 2016; Cavelier et al., 2017). Reprogramming of differentiated cells into pluripotent stem cells is achieved using Sox2 (Takahashi et al., 2007). Notably, an anti-Sox2 antibody aids in identifying oyster stem cells, covering chromatin in oyster gonia. In *C. gigas*, Sox2 is present in gonad ducts during the first sexual cycle, indicating its role in proliferation (Franco et al., 2008; Jemaà et al., 2014; Patra et al., 2015; Nuurai et al., 2016; Cavelier et al., 2017). Sox2 is distributed in a pattern similar to fish gonial proliferation, further supported by phylogenetic analysis identifying SoxB1 as a sea urchin homolog (Wei et al., 2011; Mashanov et al., 2015).

The orthologs of C-Myc and Sox2 are found in adult stem cell lines from cnidarians like Hydra, *C. hemisphaerica*, and planula larvae, indicating their involvement in proliferation (Pennati et al., 2019). *Botryllus schlosseri*, a model for pluripotency, also contains orthologs of Yamanaka factors, including Sox2 and c-Myc, hinting at their importance in stemness (Vanni et al., 2022).

In sea stars, Sox2 plays a role in nervous system regeneration, with Sox2-positive cells generating neural precursors (Zheng et al., 2022).

In the scallop *Chlamys farreri*’s the expression of Sox2 (Cf-Sox2) is observed during embryogenesis, resembling the pattern seen in mammals, implying its role in early development (Liang et al., 2015). Furthermore, the expression pattern of *Cf-sox2* was investigated in scallop *C. farreri* using the whole mount *in situ* hybridization technique. Positive hybridization signals were observed in the central region of unfertilized oocytes and fertilized eggs, and subsequently, strong signals were dispersed throughout the embryos from the 2-cell stage to gastrula. The observed expression pattern of *Cf-sox2* during embryogenesis resembled that of mammalian sox2, suggesting that *Cf-Sox2* likely regulates the early development of *C. farreri* (Liang et al., 2015).

In scallops, *Cf-*Sox2 is linked to spermatogenesis and testis development, influencing apoptosis and proliferation (Liang et al., 2019).

The genes *LsSox2* and *LsSox9*, homologs to the genes found in *Lutraria sieboldii*, exhibit differential expression in the ovary and testis, suggesting roles in embryonic and gonadal development (Lu et al., 2022). In the freshwater bivalve *Anodonta woodiana*, upregulated AwSox2 relates to apoptosis of spermatogonial stem cells and enhanced immune defense (Xia et al., 2020).

*Echinococcus multilocularis* exhibits dynamic Sox2 expression in germinative cells, which decreases upon germinative cell depletion (Cheng et al., 2017). In *Mulinia lateralis*, SOX2, FOXZ, HSFY, FOXL2, and HES1 have been identified offering insights into gonadal development and sex differentiation (Li et al., 2022). Furthermore, the regulatory effect of Oct4 and Sox9 on vitellogenesis-inhibiting hormone (SpVih) in mud crab gonadal development has been established (Liao et al., 2020). These findings collectively emphasize the significance of Sox2 in diverse developmental and regulatory processes across various organisms.

#### 3.1.17 Telomerase activity

Stem cells possess remarkable self-renewal abilities, which contribute to their immortality or prolonged existence. The maintenance of chromosomal telomeres and the presence of telomerase are vital for cellular immortality (Blackburn, 1991). Higher telomerase activity in stem cells and immortal cells indicates enhanced self-renewal potential (Morrison et al., 1996). This concept is exemplified in the colonial ascidian *B. schlosseri*, where telomerase and telomere length influence self-renewal (Laird and Weissman, 2004), and in shrimp tissues where telomerase activity predicts proliferation (Lang et al., 2004). Telomerase activity can be quantified using the telomeric repeat amplification protocol (TRAP) (Kim et al., 1994), which is extensively used to study various cell types (Belair et al., 1997). For instance, in *B. schlosseri*, telomerase activity was measured using a real-time quantitative protocol (Laird et al., 2005).

Notably, telomerase activity in shrimp lymphoid tissue increases during cultivation, peaking at 20 days (Lang et al., 2004). Immortality in stem cells comes from long telomeres and sustained telomerase activity, which is a widespread phenomenon across the animal kingdom (Traut et al., 2007). The colonial ascidian *Botrylloides* exhibits heightened telomerase activity linked to its regenerative capacity (Durham et al., 2016).

Telomerase expression was discovered in the sand scallop *Euvola ziczac* across developmental stages and adult tissues (Owen et al., 2007). Telomerase is present in various organisms, including marine species, and it safeguards telomere stability and cellular division (Greider, 1990; Campisi et al., 2001; Forsyth et al., 2002). High levels of BrdU incorporation, PCNA expression, and telomerase activity in *H. perleve*’s archaeocytes demonstrate their impressive proliferation potential. In primary cultures lasting 4 days, the purified archaeocytes exhibit a substantial 2.5-fold increase in total cell number, highlighting the potential for developing sponge cell cultures to produce valuable sponge-derived drugs (Sun et al., 2007).

In the case of *Arctica islandica*, telomerase activity and telomere lengths remain constant across ages and tissues, contributing to its exceptional lifespan (Gruber et al., 2014). Telomerase activity is detected in corals and their symbiotic algae, contributing to growth and reproductive adaptation (Zielke and Bodnar, 2010).

*Diplosoma listerianum* demonstrates extensive cell proliferation, with higher telomerase activity in bud tissue than adult tissue (Sköld et al., 2011). Telomerase functions in yeast *Saccharomyces cerevisiae* during S-phase, coinciding with chromosome end replication (Luciano et al., 2012).

Activation of telomerase in *Penaeus monodon* lymphoid cells could overcome cellular aging barriers for *in vitro* transformation (Jayesh et al., 2016). Lifespan differences in sea urchins, *Strongylocentrotus franciscanus* and *Lytechinus variegatus*, do not correlate with age-related telomere shortening (Francis et al., 2006). Daphnia species, *D. pulex* and *D. pulicaria*, exhibit no age-related decline in telomere length or telomerase activity, suggesting other factors influence their distinct lifespans (Schumpert et al., 2015).

#### 3.1.18 Vasa

The vasa gene family products are widely used as molecular markers for PGCs, encoding a conserved RNA-dependent helicase expressed in the germ line (Extavour and Akam, 2003). Vasa presence has been confirmed in various bivalve species through confocal microscopy (Milani et al., 2015; Milani et al., 2017).

Additionally, Vasa expression in Hydra, Hydractinia, polychaeta, and planarians supports the origin of germ cells from primitive totipotent stem cells (Shibata et al., 1999; Mochizuki et al., 2001; Rebscher et al., 2007; Rinkevich et al., 2009). In Hydractinia, vasa protein is found in multipotent or totipotent stem cells generating somatic and germ cells (Frank et al., 2009). Vasa’s role in germline cell formation and maintenance is established in *Drosophila*, *Caenorhabditis*, *Xenopus*, and zebrafish (Mochizuki et al., 2001; Fabioux et al., 2004a), making it a valuable marker for investigating totipotent stem cells and germline development.

The presence of Vasa-related genes in Rhizocephalans, such as *Polyascus polygenea*, *Clistosaccus paguri*, and Athelgis takanoshimensis, was studied (Shukalyuk et al., 2007). Co-expression of Vasa with PL10, Oct4, and Bl-piwi was observed in germ line derivatives and somatic tissues of *B. schlosseri* (Extavour et al., 2005). Nanomia bijuga showed co-expression of Vasa-1, PL10, piwi, nanos-1, and nanos-2 in interstitial stem cells of young zooid buds (Siebert et al., 2015).

In *B. schlosseri*, Vasa, PL10, and Oct4 co-expression identified PGC-like cells during embryonic and colony development (Rosner et al., 2009). In some cases, Vasa expression extends beyond germ cells. Vasa is found in somatic tissues of *P. dumerilii*, and in segmental tissues of Tubifex tubifex (Zelada González, 2005; Oyama and Shimizu, 2007; Rebscher et al., 2007). Acropora tenuis displays Vasa’s role in germline regulation throughout the oogenic cycle (Tan et al., 2020).

The study of *A. virens* investigates the Vasa, PL10, and Piwi genes, and their role in maintaining undifferentiated states in PGCs and multipotent stem cells (Kozin and Kostyuchenko, 2015). The Vasa orthologue in *C. gigas* is found only in germline cells (Fabioux et al., 2004b), while Mb vasa is identified in the testis of Maja brachydactyla, confirming its expression in the gonadal (Simeó et al., 2011). These findings collectively enhance our understanding of germ cell development and totipotent stem cells across various invertebrate species.

### 3.2 Applied techniques for MISC identification

Our -efforts to unlock the secrets of stem cells is guided by a wide assortment of techniques, each contributing a unique perspective to the puzzle. These techniques help us to unravel the multifaceted roles of stem cells, encompassing their involvement in developmental pathways, the stability of tissues, and the intricate mechanisms underlying diseases. By artfully combining these methods, researchers can comprehensively study stem cell populations, and gain a deeper comprehension of their functions. These versatile methodologies extend their utility not only for studying invertebrate but also for exploring mammalian systems studies. Some of the common techniques include flow cytometry, immunofluorescence/immunocytochemistry, *in situ* hybridization, Real-time RT-PCR, and western blotting. In the following, we embark on a detailed journey through these techniques, to uncover the valuable insights they provide (Tables 2, 3).

**TABLE 2:** Diversity of marine stem cell markers: comparative study across different marine organisms using various methods.

Marker	Sample	The homologs of mammalian marker	Method	Ref
Aldehyde dehydrogenase	*Fenneropenaeus chinensis*	ALDH1-like	Flow cytometry	Jiang et al. (2009)
	*Limulus polyphemus*			Dragolovich and Pierce (1994)
	*Crassostrea virginica*			Perrino and Pierce (2000)
	*Polyandrocarpa misakiensis*			Storms et al. (1999)
Alkaline phosphatase	*Polyascus polygenea*	ALP	Immunofluorescence	Isaeva et al. (2003)
	Pomatoceros lamarckii			Szabo and Ferrier (2014)
	*Crassostrea giga*			Cavelier et al. (2017)
Bmi1	*Holothuria glaberrima*	BMI1	ISH, RT-PCR	Mashanov et al. (2017b)
Brdu	*Hymeniacidon perleve*	BrdU	IHC	Sun et al (2007)
	*Hydractinia echinate*			Müller et al. (2004)
	*Clytia hemisphaerica*			Denker et al. (2008)
c-Myc	*Holothuria glaberrima*	c-Myc	ISH, RT-PCR	Mashanov et al. (2015)
Edu	*Aeolosoma viride*	EdU	IHC	Chen et al. (2020a)
	*Pristina leidyi*			Bely and Sikes (2010)
	*Playtnereis dumerilli*			Rebscher et al. (2012), Ćorićr et al. (2022)
	*Nematostella vectensis*			Amiel et al. (2015)
	*Ptychodera flava*			Luttrell et al. (2018)
	*Fimbriaphyllia ancora*			Chiu et al. (2021)
	*Pleurobrachia pileus*			Alié et al. (2011)
Grimp	*Enchytraeus japonensis*		ISH, RT-PCR	Takeo et al. (2009), Yoshida-Noro and Tochinai (2010)
Klf	*Holothuria glaberrima*	Klf1/2/4	ISH, RT-PCR	Mashanov et al. (2015)
	*Mnemiopsis leidyi*	Klfs		Presnell (2017)
	*Schmidtea mediterranea*	klf4l		Issigonis et al. (2022)
	*Apostichopus japonicus*	Aj-klf13		Chen et al. (2020b)
	*Strongylocentrotus purpuratus*	klf2/4,klf3/8/12, klf7,klf11, klf13, klf15		Materna et al. (2006)
Lgr4/5/6	*Holothuria glaberrima*	Lgr4/5/6	ISH, RT-PCR	de Lau et al. (2014)
Nanog	*Strongylocentrotus purpuratus*	Nanog	IHF, RT-PCR	Ballarin et al. (2022)
Nanos	*Pristina leidyi*	*Nanos*	ISH	Özpolat and Bely (2015)
	*P. ornatus*		RT-PCR	Helluy and Beltz (1991)
	*Helobdella robusta*			Kang et al. (2002)
	*Platynereis dumerilii*			Zelada González (2005), Rebscher et al. (2007)
	*Ilyanassa obsolete*			Rabinowitz et al. (2008)
	*Patinopecten yessoensis*			Liu et al. (2022)
	*Haliotis asinine*	*HasNanos*		Kranz et al. (2010)
	*Hydractinia echinata*	*Nanos*	ISH	Millane et al. (2011)
Oct-4	*Hydractinia stolons*	*Oct1/2*	ISH	Eddy (1976)
	*Holothuria glaberrima*	Oct1/2/11	ISH, Real-time RT-PCR	Mashanov et al. (2015)
	*Scylla paramamosain*	Oct4	Real-time RT-PCR	Liao et al. (2020)
	*Botryllus schlosseri*		IHC	Rosner et al. (2009)
	*Paracentrotus lividus*	Oct1/Oct2	ISH	Range and Lepage (2011)
PCNA	*Hymeniacidon perleve*	PCNA	IF	Sun et al. (2007)
	*Botrylloides leachi*		IHC	Hyams et al. (2017)
	*Montipora foliosa*		RT-PCR	D’Angelo et al. (2012)
	*Crassostrea gigas*	CgPCNA	RT-PCR	Yu et al. (2022)
	*Acropora pulchra*	PCNA	RT-PCR	D’Angelo et al. (2012)
Piwi	*Botrylloides violaceus*	Piwi	IHC, Western blot	Brown and Swalla (2012)
	*Botrylloides leachi*		ISH, IHC	Vickers (2017)
	*Podocoryne carne*	Cniwi		Seipel et al. (2003)
	*Botrylloides schlosseri*	Piwi		Rabinowitz and Rinkevich (2011)
	*Capitella teleta*		ISH	Giani et al. (2011)
	*Enchytraeus japonensis*			Yoshida-Noro and Tochinai (2010)
	*Alitta virens*			Kozin and Kostyuchenko (2015)
	*Euphyllia ancora*			Shikina et al. (2012), Shikina et al. (2015)
	*Platynereis dumerilii*			Weigert et al. (2013)
	*L. sanguineus*	Ls-piwi2/3		Xu and Sun (2020)
Pl 10	*Ephydatia fluviatilis*	Pl 10	RT-PCR	Mochizuki et al. (2001)
	*Hydra magnipapillata*		RT-PCR, ISH	Mochizuki et al. (2001)
	*Alitta virens*		ISH	Kozin and Kostyuchenko (2015)
	*Capitella teleta*		IF	Seaver and de Jong (2021)
	*Pleurobrachia pileus*		RT-PCR, ISH	Alié et al. (2011)
Sox2	*Clytia hemisphaerica*	Sox2	ISH	Pennati et al. (2019)
	*Botryllus schlosseri*	SoxB1	RT-PCR	Vanni et al. (2022)
	*Patiria miniate*	Sox 4+	IF	Zheng et al. (2022)
	*Chlamys farreri*	*Cf-Sox2*	RT-PCR	Liang et al. (2015), Liang et al. (2019)
	*L. sieboldii*	*LsSox2/9*	ISH, Real-time RT-PCR	Lu et al. (2022)
	*Anodonta woodiana*	*AwSox2*		Xia et al. (2020)
	*Echinococcus multilocularis*	Sox2	ISH	Cheng et al. (2017)
	*Scylla paramamosain*	Sox9		Liao et al. (2020)
	*Holothuria glaberrima*	SoxB1	ISH, Real-time RT-PCR	Mashanov et al. (2015)
Telomerase activity	*Botryllus schlosseri*		TRAP	Laird and Weissman (2004), Laird et al. (2005)
	*Euvola ziczac*		ISH	Owen et al. (2007)
	*A. islandica*		TRAP	Zielke and Bodnar (2010)
	*Hymeniacidon perleve*		TRAP	Sun et al. (2007)
	*Diplosoma listerianum*		RT-PCR	Sköld et al. (2011)
	*Strongylocentrotus franciscanus*		ISH, RT-PCR	Francis et al. (2006)
	*Lytechinus variegatus*			Francis et al. (2006)
	*D. pulex*			Schumpert et al. (2015)
Vasa	*Polyascus polygenea*	Vasa	ISH, RT-PCR	Shukalyuk et al. (2007)
	*Clistosaccus paguri*			Shukalyuk et al. (2007)
	*Athelgis takanoshimensis*			Shukalyuk et al. (2007)
	*Botryllus schlosseri*			Extavour et al. (2005)
	*Tubifex tubifex*			Oyama and Shimizu (2007)
	*Acropora tenuis*			Tan et al. (2020)
	*Alitta virens*			Kozin and Kostyuchenko (2015)
	*Ephydatia fluviatilis*			Mochizuki et al. (2001)
	*Hydra magnipapillata*			Mochizuki et al. (2001)

**TABLE 3:** Benefits and limitations of methods used for identifying marine stem cell markers.

Method	Type of identification	Advantage	Disadvantage	Supplement explanation	References
Flow cytometry	Phenotypic	⁃ High sensitivity ⁃ High speed analysis ⁃ Reveals the phenotype of individual cells ⁃ Examines several markers simultaneously ⁃ Can be used to isolate specific populations (when equipped with a cell sorter) ⁃ Gating Can used to eliminate dead cells from analysis	⁃ Requires expensive equipment ⁃ Requires skilled operator ⁃ Potential cross reactivity when using several antibodies ⁃ Potential autofluorescence ⁃ Cell sorting by FACS can be time consuming and expensive	Stem cells can be identified and isolated from mixed cell population by staining the cells with specific antibody markers (fluorescent antibody cell sorting) by using a flow cytometer	Wlodkowic et al. (2011), Wlodkowic et al. (2013)
Immunofluorescence/Immunocytochemistry	Phenotypic	⁃ Reveals localization of marker proteins ⁃ Can assess multiple markers simultaneously ⁃ More efficient than Western blot analysis ⁃ Can use live or fixed cells	⁃ Requires specialized equipment ⁃ Potential for cross-reactivity when using multiple antibodies ⁃ Potential autofluorescence Photobleaching ⁃ More time consuming than flow cytometry		Lichtman and Conchello (2005), Odell and Cook (2013)
*In situ* hybridization (ISH)	Phenotypic	⁃ providing valuable insights for anatomical interpretations ⁃ identify cells by their unique mRNA content ⁃ accurately localize mRNA at the cellular level in intricate tissues	⁃ false-negative results can occur due to RNA loss or probe failure	By localizing gene sequences and visualizing gene expression products in their original context, ISH maintains cellular integrity in heterogeneous tissues, providing valuable insights for anatomical interpretations	Jin and Lloyd. (1997)
Real-time RT-PCR	Phenotypic	⁃ Detects early changes in marker expression induced by differentiation ⁃ Analyze several markers simultaneously ⁃ High sensitivity and specificity	⁃ Yields the average marker expression of a population ⁃ Does not reveal heterogeneity	A semi-quantitative method to detection specific gene transcripts of stem and germ cells	Maddocks and Jenkins. (2017)
Western Blot	Phenotypic	⁃ High sensitivity ⁃ High specificity ⁃ Does not require specialized equipment	⁃ Does not reveal heterogeneity of cell population ⁃ Low throughput		Laemmli. (1970)

#### 3.2.1 Flow cytometry

Flow cytometry, a potent analytical tool, enables for the simultaneous assessment of multiple cell markers within single cells. This method employs fluorescently labeled antibodies that bind to cell surface or intracellular markers. Subsequently, the labeled cells flow through a cytometer, which measures emitted fluorescence to quantify marker expression. Researchers utilize flow cytometry to delineate diverse cell populations based on marker profiles, making it useful for investigating heterogeneous groups, and identifying rare subsets (Schachtele et al., 2020). Additionally, this technique can be used for cell proliferation and cycle analysis (Wlodkowic et al., 2011; Wlodkowic et al., 2013). Flow cytometry dissects cell functions via surface and intracellular staining, hinges on cell surface antigens for cell type identification. Moreover, flow cytometry provides deeper insights by concurrently staining both cell surface and intracellular contents within single cells (Adan et al., 2017).

Flow cytometry is a valuable tool for both researchers and clinicians, facilitates identifying, characterizing, and isolating stem and progenitor cells for research and potential clinical applications (Adams et al., 2009). Multicolor flow cytometry enables the simultaneous examination of diverse stem cell markers (Schachtele, Clouser and Aho). This method has been effectively revealed stem cells in various organisms, including post-blastema tissue analysis in regenerating planarians, shedding light on neoblasts, or planarian stem cells (Ermakov et al., 2012).

In the case of *Eupentacta fraudatrix*, flow cytometry unveiled quantitative variations in coelomic fluid cells post-evisceration over 24 h. Notable dynamics emerged between minimally differentiated juvenile cells and well-differentiated cells during regeneration. The juvenile coelomocyte population increased, while differentiated cell quantity decreased, establishing a reciprocal pattern (Zavalnaya et al., 2020).

#### 3.2.2 Immunofluorescence: immunocytochemistry/immunohistochemistry

Immunofluorescence, including immunocytochemistry and immunohistochemistry, employs antibodies to visualize specific markers within cells. Cells fixed on slides are first incubated with primary antibodies binding to target markers. Subsequently, fluorescently labeled secondary antibodies recognize the primary antibodies, producing a fluorescent signal at marker locations. This technique provides spatial insights into marker expression and subcellular localization (Odell and Cook, 2013). To maintain signal quality, samples must be shielded from light when not in use, ensuring reliable results (Lichtman and Conchello, 2005). Signal strength depends on antibody quality, sample handling, and secondary antibody detection (Odell and Cook, 2013).

An innovative approach uses immunocytochemistry to identify my-Vasa-positive germ cells in Yesso scallops, shedding light on reproductive cycle dynamics and shellfish hatcheries (Mokrina et al., 2021).

Another study utilizes immunocytochemistry with vasa antibodies, reverse transcription, and polymerase chain reaction to detect germline cell markers, providing a methodological basis for investigating oogonia in sea urchins (Yakovlev et al., 2010). Microfilament elongation towards the vegetal hemisphere is observed using time-lapse video and fluorescent labeling, emphasizing their role in germ plasm formation (Prodon et al., 2009). Immunocytochemistry with anti-Vasa antibodies reveals germ plasm and nuage material aggregation in chaetognaths (Carré et al., 2002).

Fluorescent and non-fluorescent recombinant proteins are effectively employed to track events in sea urchin post-fertilization. Microinjection of proteins and dyes into unfertilized eggs, observed via fluorescence, confocal, or two-photon microscopy, provides insights into the first cell cycle (Kisielewska and Whitaker, 2014).

#### 3.2.3 *In situ* hybridization

*In situ* hybridization (ISH) seamlessly merges molecular biology and histochemical techniques to reveal gene expression within tissues and cytological samples. By pinpointing gene sequences and visualizing their products within their native context, ISH preserves cellular integrity in complex tissues, yielding anatomical insights (Jin and Lloyd, 1997). ISH uniquely identifies cells based on mRNA content, ensuring accuracy compared to potential false positives in immunostaining (Jin and Lloyd, 1997). A significant advantage is ISH’s ability to precisely localize mRNA at a cellular level, enhancing our understanding of intricate tissues and complementing other molecular methods. It is worth noting that false negatives can arise from RNA loss or probe issues, especially in retrospective paraffin-embedded studies (Jin and Lloyd, 1997).

Vasa orthologs serve as dependable markers for germline identification across species. In adult and juvenile pearl oysters (*Pinctada fucata*), ISH of the vasa ortholog (povlg1) unveils immature germ cells undetectable with traditional staining, benefiting pearl quality enhancement (Sano et al., 2015). Similarly, in *L. sanguineus*, ISH exposes distinct ovarian expression of Ls-piwi1 compared to absent Ls-piwi2 and Ls-piwi3 (Xu and Sun, 2020).

For another annelid, adult *Myzostoma cirriferum*, ISH highlights exclusive Piwi-like gene (Mc-Piwi1, Mc-Piwi2) presence in gonadal stem cells, excluding the posterior growth zone or somatic stem cells (Weigert et al., 2013). In *C. hemisphaerica*, a hydrozoan cnidarian, ISH reveals germ cell marker expressions (Vasa, Nanos1, Piwi, PL10) in multipotent interstitial cells during larval and adult medusa stages (Leclère et al., 2012). Focusing on *F. chinensis*, ISH confirms Fc-vasa transcript localization in spermatogonia and oocytes, illuminating Fc-Vasa’s role in germ-line development and potential as a marker (Zhou et al., 2010).

#### 3.2.4 Real-time RT-PCR

The quantitative polymerase chain reaction (Q-PCR) is a valuable technique for assessing gene expression levels by measuring PCR product quantities in real-time. This method, also known as real-time PCR or quantitative reverse transcriptase PCR (RT-PCR), helps determine gene expression under specific conditions (Maddocks and Jenkins, 2017). In the study of prostate cancer cell lines and embryonic stem cells, reprogramming factor expression was evaluated using semi-quantitative RT-PCR (244). *Fc-vasa* exhibited gonadal-specific expression in Chinese shrimp, *F. chinensis*, with levels changing during gonadal development. Additionally, *Fc-PL10a* displayed widespread expression in examined tissues (Bae et al., 2010; Zhou et al., 2010).

Vasa is used as a molecular marker for PGCs visualization in various species. In tongue sole (*Cynoglossus semilaevis*), the vasa gene characterization identified to have three transcripts: vas-l, vas-m, and vas-s. Quantitative real-time PCR analysis has highlighted prevalent gonadal expression, particularly for vas-s during embryonic development (Wang et al., 2014). In *Mytilus coruscus*, gonad-specific genes and miRNAs were identified through transcriptome sequencing. The qRT-PCR analysis revealed differential expression, while miRNA targeting was confirmed through luciferase assays (Wang et al., 2022).

In the spider crab (*Maja brachydactyla*), the Mb vasa expression was examined using quantitative PCR during early post-embryonic development, showing fluctuations with developmental stages (Simeó et al., 2011). Furthermore, RNA interference targeting oyster vasa-like gene (*Oyvlg*) in *C. gigas* led to reduced germ cell proliferation and meiosis arrest (Fabioux et al., 2009).

In *Botrylloides leachi*, Mitomycin C treatments and siRNA knockdown of *Bl-Piwi* revealed its importance in regeneration and colony adaptation (Rinkevich et al., 2010). Analysis of BS-Vasa in Botryllus schlosseri demonstrated its presence in colony cells beyond germ cell lineages (Rosner et al., 2009).

#### 3.2.5 Western blot

Western blotting is a sensitive technique employed to detect specific proteins or antibodies within complex mixtures that interact with discrete antigens (Laemmli, 1970). This method’s heightened sensitivity and specificity make it ideal for identifying low-concentration proteins. It allows the separation of intricate mixtures into individual components based on molecular weight and post-translational modifications, facilitating the identification of low-reactivity antigens (Kurien and Scofield, 2009).

However, it is important to acknowledge Western blotting’s limitations. Relying solely on a molecular weight ladder for comparison might lead to inaccurate conclusions. This is particularly pertinent for post-translational modification proteins, as the predicted molecular weight from the genome sequence might not precisely correspond to the actual gene product. Furthermore, the use of sodium dodecyl sulfate polyacrylamide gel electrophoresis (SDS-PAGE) could irreversibly denature all proteins in the lysate, potentially affecting the target protein’s detection (Algenäs et al., 2014).

In a study on Baltic Sea blue mussels, the impact of exposure to various doses of benzo [a]pyrene (BaP) for 3 days was investigated through the analysis of protein biomarkers. Notably, mussels exposed to the lowest dose, corresponding to the minimum reported dose for DNA adduct formation in their gills, exhibited significant changes in protein expression. Western blot analysis quantified the upregulated expression of proliferating cell nuclear antigen (PCNA). Conversely, immunocytochemistry analysis of the 5-bromo-deoxyuridine (BrdU) staining pattern revealed no substantial changes. These findings suggest that the PCNA response likely indicated non-proliferative activity, potentially linked to DNA damage (Prevodnik et al., 2007). Additionally, in order to gain insights into bivalve germ line development, VASA ortholog-specific antibodies were used to investigate different species, including Ruditapes philippinarum, Scapharca inaequivalvis, *C. gigas*, and *Mya arenaria*. Western blot analysis confirmed the immunoreactivity of the anti-VASA antibodies, displaying distinct bands in each species, with varying molecular weights. It`s worth noting that *M. arenaria*, like Ruditapes philippinarum, is reported as gonochoric (Milani et al., 2016).

## 4 Current limitations/challenges

Despite significant advancements in the study of marine invertebrate stem cells, several limitations and challenges persist.

1) Genetic Tools: The lack of comprehensive genetic tools for many marine invertebrates limits the ability to perform detailed functional analyses. While techniques such as RNAi and CRISPR-Cas9 are promising, their application is not yet widespread across all species.
2) Marker Identification and Validation: Research on marine invertebrate stem cells requires the identification of more markers to distinguish between differentiation levels of cells in different species. This will allow for the identification and isolation of pluripotent/totipotent stem cells. Validating these markers to ensure they indicate stem cell activity, rather than simply being orthologous to vertebrate markers, is crucial. This issue is directly associated with the problem of de-differentiation. In several cases, such as ascidian palleal budding or starfish regeneration, it cannot be excluded that cells from injured tissues or budding areas de-differentiate, adopting a stem cell phenotype capable of forming a bud primordium or a blastema. Unlike in mammals, this spontaneous de-differentiation under certain circumstances in invertebrates requires additional investigation for a deeper understanding.
3) Species-Specific Protocols: Each invertebrate species may require specific protocols for successful gene perturbation, making it difficult to generalize findings across different taxa.
4) Functional Studies on Adult Organisms: Functional experiments on adult marine invertebrates are often more challenging than on their larval or juvenile stages due to the complexity and resilience of adult tissues.
5) Technical limitations: Further efforts are needed to foster cooperation among organizations working on aquatic invertebrate stem cells. Enhanced collaboration could increase the use of invertebrate adult stem cells in biological research and help overcome technical problems that have hindered the achievement of stable stem cell cultures from aquatic invertebrates.

To advance our understanding of invertebrate stem cells, it is crucial to leverage existing functional studies while acknowledging the current limitations. By focusing on the available gene perturbation assays and their findings, we can highlight the unique aspects of invertebrate stem cells and pave the way for future research that addresses the current challenges. Additionally, developing and refining genetic tools for a broader range of invertebrate species will enhance our ability to perform comprehensive functional analyses and uncover the intricate biology of these fascinating organisms.

## 5 Conclusion

**FIGURE 2:**
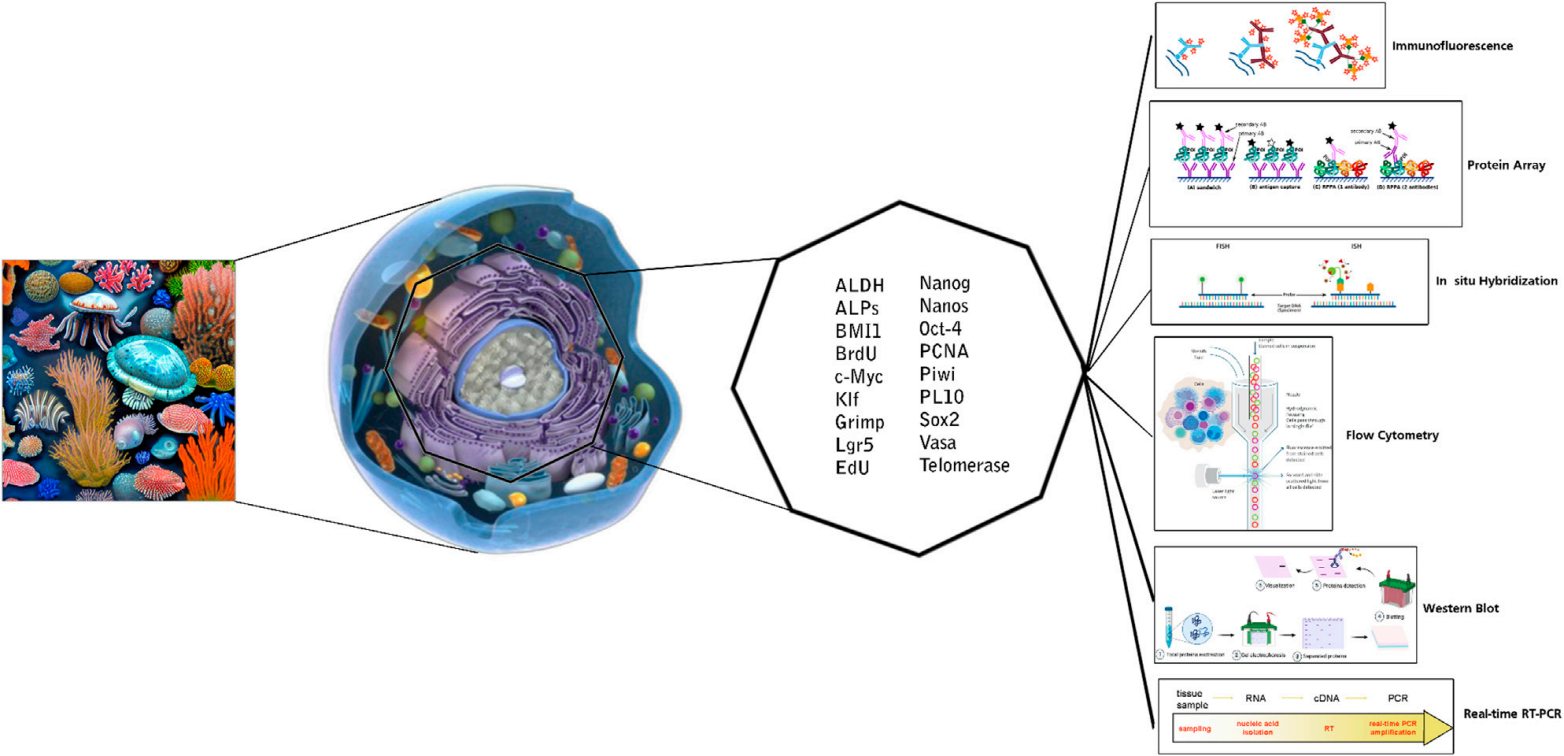
Markers and applied techniques for *in vitro* detection of marine invertebrate stem cells.

This study underscores the critical role of marker expression analysis in advancing our understanding of marine invertebrate stem cells. The deployment of specific markers and sophisticated molecular techniques, such as flow cytometry, immunocytochemistry, protein arrays, and immunoblotting, offers profound insights into the characterization and identification of these stem cells (Figure 2). Through detailed investigation of marker expression patterns, researchers can explore the potential of marine invertebrate stem cells for applications in regenerative medicine, drug discovery, and the broader study of these unique organisms. The integration of these techniques with advanced methods like electron microscopy provides a comprehensive view of the distinctive properties of marine invertebrate stem cells. Looking ahead, ongoing advancements in marker detection, coupled with the integration of omics approaches and computational tools, promise to further refine our understanding of stem cell differentiation, cell heterogeneity, and therapeutic applications. These developments will enhance our ability to analyze individual cell profiles and marker expression across cell populations.

Overall, this article highlights the significance of marker expression analysis and the application of advanced techniques in marine invertebrate stem cell research. By offering valuable insights while circumventing ethical issues associated with human cell sources, these studies pave the way for future breakthroughs in regenerative medicine and marine biotechnology. Continued research in this field will undoubtedly lead to the discovery of novel markers, a deeper understanding of cellular mechanisms, and significant advancements in the application of marine invertebrate stem cells.
